# Human Cases of Highly Pathogenic Avian Influenza A**(**H5N1**)** — California, September–December 2024

**DOI:** 10.15585/mmwr.mm7408a1

**Published:** 2025-03-13

**Authors:** Sophie Zhu, Kathleen Harriman, Caterina Liu, Vit Kraushaar, Cora Hoover, Kyoo Shim, Sharon I. Brummitt, Jocelyn Limas, Kathleen Garvey, Jennifer McNary, Nina J. Gao, Rahil Ryder, Brandon Stavig, Jeffrey Schapiro, Christina Morales, Debra A. Wadford, Holly Howard, James Heffelfinger, Rebecca Campagna, Esmeralda Iniguez-Stevens, Hamed Gharibi, Denise Lopez, Laura Esbenshade, Paula Ptomey, Kavita K. Trivedi, Jade A. Herrera, Joanna Locke, Nicholas Moss, Paul Rzucidlo, Kimberly Hernandez, Minhphuong Nguyen, Simon Paul, Justin Mateo, Carlos Del Carmen Luna, Yer Chang, Maria Rangel, Keiryl DeLeon, Aisha Masood, Thea Papasozomenos, Payeng Moua, Katie Reinhart, Krista Kniss, C. Todd Davis, Marie K. Kirby, Erica Pan, Erin L. Murray, Los Angeles County H5 Response Team, California Department of Public Health H5 Laboratory Response Team, Los Angeles County H5 Response Team, Annabelle de St. Maurice, Eric El-Tobgy, Nicole Green, Allison Joyce, Cristin Mondy, Taylor Mundt, Heidi Ransohoff, Shayra Sanchez, Elizabeth Traub, California Department of Public Health H5 Laboratory Response Team, Matthew Bacinskas, John Bell, Cynthia Bernas, Brandon Brown, Jahara Cayabyab, Alice Chen, Jesse Elder, Shiffen Getabecha, Carol Glaser, Olena Gomez, Bianca Gonzaga, Ydelita Gonzales, Hugo Guevara, April Hatada, Katya Ledin, Deidra Lemoine, Adrienne Macias, Sergio Martinez-Paredes, Blanca Molinar, Tasha Padilla, Chao-Yang Pan, Kiana Pattni, Rolando Ramirez, Kao Saechao, Estela Saguar, Maria Salas, Ioana Seritan, Anthony Tran, Cindy Wong, Chelsea Wright

**Affiliations:** ^1^Epidemic Intelligence Service, CDC; ^2^California Department of Public Health; ^3^Tulare County Department of Health and Human Services, Visalia, California; ^4^Alameda County Public Health Department, San Leandro, California; ^5^Kern County Public Health, Bakersfield, California; ^6^Madera County Department of Public Health, Madera, California; ^7^Merced County Public Health, Merced, California; ^8^Fresno County Department of Public Health, Fresno, California; ^9^San Joaquin County Public Health Services, Stockton, California; ^10^Stanislaus County Health Services Agency, Modesto, California; ^11^Influenza Division, National Center for Immunization and Respiratory Diseases, CDC.; Los Angeles County Department of Public Health; Los Angeles County Department of Public Health; Los Angeles County Department of Public Health; Los Angeles County Department of Public Health; Los Angeles County Department of Public Health; Los Angeles County Department of Public Health; Los Angeles County Department of Public Health; Los Angeles County Department of Public Health; Los Angeles County Department of Public Health; California Department of Public Health; California Department of Public Health; California Department of Public Health; California Department of Public Health; California Department of Public Health; California Department of Public Health; California Department of Public Health; California Department of Public Health; California Department of Public Health; California Department of Public Health; California Department of Public Health; California Department of Public Health; California Department of Public Health; California Department of Public Health; California Department of Public Health; California Department of Public Health; California Department of Public Health; California Department of Public Health; California Department of Public Health; California Department of Public Health; California Department of Public Health; California Department of Public Health; California Department of Public Health; California Department of Public Health; California Department of Public Health; California Department of Public Health; California Department of Public Health; California Department of Public Health; California Department of Public Health; California Department of Public Health

**Summary**

**What is already known about this topic?**

Persons with occupational exposure to highly pathogenic avian influenza (HPAI) A(H5N1) virus–infected dairy cattle are at increased risk for infection.

**What is added by this report?**

During September 30–December 24, 2024, a total of 38 persons received a positive test result for HPAI A(H5N1) viruses in California; 37 were dairy farm workers with occupational exposure to sick cows. One, a person aged <18 years with an undetermined exposure, was the first pediatric patient detected with influenza A(H5) infection in the United States.

**What are the implications for public health practice?**

Public health agencies should investigate influenza-like illness or conjunctivitis in workers with occupational exposure to animals infected with HPAI A(H5N1) virus. Thorough investigations of all human HPAI A(H5N1) virus infections are necessary to identify potential exposure sources, including monitoring the virus for concerning genetic changes that indicate the potential for person-to-person transmission.

## Abstract

Persons who work closely with dairy cows, poultry, or other animals with suspected or confirmed infection with highly pathogenic avian influenza (HPAI) A(H5N1) viruses are at increased risk for infection. In September 2024, the California Department of Public Health was notified of the first human case of HPAI A(H5N1) in California through monitoring of workers on farms with infected cows. During September 30–December 24, 2024, a total of 38 persons received positive test results for HPAI A(H5N1) viruses in California; 37 were dairy farm workers with occupational exposure to sick cows, and one was a child aged <18 years with an undetermined exposure, the first pediatric HPAI A(H5N1) case reported in the United States. All patients had mild illness. The identification of cases associated with occupational exposure to HPAI A(H5N1) viruses on dairy farms highlights the continued risk for persons who work with infected animals. The pediatric case was identified through routine surveillance. Given recent increases in the prevalence of HPAI A(H5N1) viruses among some animal populations, public health agencies should continue to investigate cases of HPAI A(H5N1) in humans as part of control measures, pandemic preparedness, to identify concerning genetic changes, and to prevent and detect potential human-to-human transmission of the virus. To date, no human-to-human transmission of HPAI A(H5N1) virus has been identified in the United States.

## Introduction

Novel influenza A virus infection, including highly pathogenic avian influenza (HPAI) A(H5N1) virus, is a reportable condition in California and nationally reportable to CDC.* In 2024, the California Department of Public Health (CDPH), California Department of Food and Agriculture (CDFA), local health departments (LHDs), and farms known to be affected by HPAI A(H5N1) (i.e., dairy or poultry farms with nonnegative [positive or inconclusive] A(H5) test results for cows, bulk milk, or poultry) coordinated to reduce infection risk and monitor HPAI A(H5N1) symptoms^†^ among workers. All farm owners or managers of affected farms were advised to conduct daily monitoring of workers and report symptoms consistent with HPAI A(H5N1) infection in workers who were in contact with affected animals to their LHD. When farm owners did not volunteer to do the monitoring, the LHD offered to perform monitoring of symptoms directly with workers through phone calls or text messaging. Symptomatic workers were referred for specimen collection, typically, conjunctival, nasal, nasopharyngeal, or oropharyngeal swabbing, based on symptom presentation. Targeted surveillance, which includes influenza typing and subtyping for A(H5), was performed at either a local or the state public health laboratory (PHL) for all symptomatic workers or persons with epidemiologic linkage (*1*) to HPAI A(H5N1) reported to public health officials. PHLs use the CDC Human Influenza A Subtyping Kit which detects and differentiates hemagglutinin (H) proteins as part of routine influenza surveillance. Selected local PHLs employ the CDC Influenza A(H5) Subtyping Kit to detect A(H5)^§^ Asian lineage viruses for suspected HPAI A(H5N1) cases. Presumptive positive or inconclusive A(H5) specimens were sent to CDC for confirmatory testing. This report summarizes information on human HPAI A(H5N1) cases identified in California during September 30–December 24, 2024.

## Investigation and Results

### Initial Public Health Notification and Response

On August 30, 2024, CDFA detected, and the National Veterinary Services Laboratories subsequently confirmed, HPAI A(H5N1) virus infections in cows from three dairy farms in the Central Valley region of California. In September 2024, CDPH was notified of the first human case of HPAI A(H5N1) in California through monitoring of workers on farms with infected cows. On October 3, 2024, the first two human HPAI A(H5N1) cases in California were confirmed in workers on two separate farms where infected cows were detected in September. These patients had been identified and reported by their employers to their LHD; both had conjunctivitis, and one also had a fever. Specimens from both patients tested positive for influenza A(H5) virus at a local PHL and were confirmed as HPAI A(H5N1) at CDC. LHD staff members provided guidance on isolation and offered the antiviral oseltamivir to patients and their household members. No known epidemiologic links existed between the two patients.

As of December 24, 2024, the U.S. Department of Agriculture reported 675^¶^ dairy herds with infected cows, 92 commercial flocks with infected poultry,** and 35 backyard flocks with infected poultry in California. During August 30–December 24, a total of 5,126 workers were monitored at affected farms; 170 persons from 19 local health jurisdictions received testing for influenza A(H5) through targeted surveillance. One additional patient was reported through routine surveillance and subsequently received testing at a PHL. Of the 171 persons who received testing, CDPH identified 36 confirmed cases and one probable (*1*) case of HPAI A(H5N1) among adult dairy farm workers and one confirmed case in a child aged <18 years without dairy cow or poultry exposure; 37 persons received positive test results confirmed at CDC. This activity was reviewed by CDC and CDPH, deemed research not involving human subjects, and was conducted consistent with applicable federal law and CDC policy.^††^

### Description of Human HPAI A(H5) Cases

**Human cases with exposure to dairy cows (37).** Persons with HPAI A(H5N1) infection (36 confirmed and one probable) worked at 29 unique dairy farms (Table 1). The median interval from first A(H5) virus detection in cows to the first human case on a particular farm was 7 days (range = −7 to 20 days). Worker monitoring was initiated on one unaffected farm because A(H5) virus had been detected in cows on other dairy farms owned by the same person. All patients with occupational exposure to dairy cows were aged 18–64 years (Table 2). Six patients reported underlying medical conditions. A majority (76%) worked as milkers or cared for sick cows. A majority of patients (78%) reported using personal protective equipment (PPE) at work; 25 (68%) wore gloves, 20 (54%) used eye protection (13 reported wearing goggles), 12 (32%) reported wearing boots, and six (16%) wore gowns. No patients specifically reported wearing a respirator (e.g., an N95 mask) as recommended^§§^; however, 12 (32%) reported wearing other face coverings or face masks.

**TABLE 1:** Characteristics of dairy farms with associated human highly pathogenic avian influenza A(H5N1) cases — California, September–December 2024

Farm	No. of workers monitored	No. of human cases	Days under quarantine* as of December 24, 2024	No. of days from first A(H5) virus detection in cows to first human case
A	Unknown	3	81	6
B	Unknown	2	71	11
C	Unknown	2	60	2
D	40	3	50	10
E	Unknown	1	85	6
F	30	1	95	14
G	Unknown	1	95	13
H	Unknown	1	82	6
I	Unknown	1	85	13
J	10	1	81	6
K	7	1	81	6
L	26	1	81	10
M	Unknown	1	70	7
N	Unknown	1	71	5
O	23	1	70	7
P	80	1	81	20
Q	Unknown	1	60	3
R	14	1	57	6
S	Unknown	1	53	10
T	13	1	53	10
U	Unknown	1	41	−7^†^
V	Unknown	1	39	0
W	Unknown	1	42	12
X	Unknown	1	39	11
Y	11	1	39	12
Z	33	1	39	12
AA	7	3	14	0
BB	Unknown	1	14	5
CC	Unknown	1	11	4

* Farms were quarantined until reporting no cows with signs of infection and three consecutive weekly negative tests of bulk milk; no farms with human cases were released from quarantine through December 24, 2024. Quarantine of sick cows is necessary to reduce farm-to-farm and cow-to-human transmission of highly pathogenic avian influenza A(H5N1) viruses.

^†^ Worker monitoring was initiated on farm U because A(H5) virus had been detected in cows on other dairy farms with the same owner. The virus was detected on the farm after the first human case occurred in a farm worker.

**TABLE 2:** Characteristics and laboratory results of persons with confirmed and probable highly pathogenic avian influenza A(H5N1) virus infection — California, September–December 2024

Characteristic	Confirmed and probable no. (%)
**Total**	38*
Confirmed	37 (97.4)
Probable^†^	1 (2.6)
Median age, yrs (IQR)	43 (32–49)
**Race and ethnicity (n = 37)^§^**	
White and Hispanic or Latino	24 (64.9)
Unknown race and Hispanic or Latino	13 (35.1)
**Primary language**	
Spanish	27 (71.0)
English	3 (7.9)
Unknown	8 (21.1)
**Public health laboratory test result**	
Presumptive positive	37 (97.4)
Negative	1 (2.6)
**CDC confirmatory result by testing site^¶^**	
Conjunctival swab (n = 37)	35 (94.6)
Nasal/Oropharyngeal swab (n = 29)	8 (27.6)
Nasopharyngeal swab (n = 37)	5 (13.5)
Nasal (n = 6)	2 (33.3)
Oropharyngeal (n = 4)	1 (25.0)
**Clinical signs and symptoms**	
Eye irritation or redness	37 (97.4)
Fever**	11 (28.9)
Muscle aches	13 (34.2)
Headache	10 (26.3)
Sore throat	6 (15.8)
Cough	6 (15.8)
Shortness of breath	4 (10.5)
Vomiting	2 (5.3)
Diarrhea	2 (5.3)
Fatigue	7 (18.4)
Dairy farm exposure	37 (97.4)
**Role on dairy farm (n = 37)**	
Milker	23 (62.2)
Farmhand	2 (5.4)
Other^††^	3 (8.1)
Unknown	9 (24.3)
**Unique dairy farms where cases occurred**	29
**Reported use of any personal protective equipment^§§^ at work (n = 32)**	
Yes	29 (78.4)
No	5 (13.5)
Unknown	3 (9.4)
**Patient offered oseltamivir**	
Accepted	36 (94.7)
Declined	2 (5.3)
**Hospitalized**	
Yes	0 (—)
No	38 (100)

* Table includes 37 persons with occupational exposure to infected dairy cows and one with an unknown exposure source to influenza A(H5).

^†^ https://cdn.ymaws.com/www.cste.org/resource/resmgr/position_statements_files_2023/24-ID-09_Novel_Influenza_A.pdf

^§^ Race and ethnicity not described for one person to protect privacy.

^¶^ Some cases were confirmed with more than one specimen.

** Measured or subjective fever.

^††^ Farmhands and persons in the “Other” categories were in roles with close contact with sick cows.

^§§^ Eye protection (including goggles), gloves, gown, or boots.

Patients received testing a median of 2 days (range = 0–5 days) after symptom onset. All patients had mild illness. Frequently reported signs and symptoms included eye irritation or redness (97%), muscle aches (34%), and fever (29%). Respiratory symptoms, including sore throat (16%) and shortness of breath (11%) were less commonly reported. No hospitalizations or deaths occurred, and all patients recovered. All 37 patients were offered oseltamivir; two declined (5%). No cases were identified in household contacts of patients with occupational exposure.

**Undetermined exposure source (one).** One confirmed case was detected through routine influenza surveillance in a previously healthy child who had no known contact with infected animals or humans and had not consumed unpasteurized dairy products. This patient, who had mild respiratory symptoms and otitis media but no conjunctivitis, was not hospitalized. Oseltamivir was prescribed when positive test results were received for influenza A virus. Subtyping was positive for influenza A(H5) virus.^¶¶^ The patient’s three household members also had respiratory symptoms; one developed symptoms a day before the patient, while the two other members developed symptoms concurrently. Four days after the patient’s initial testing, respiratory specimens were collected from all household members. All specimens tested negative for influenza A(H5) virus. Specimens from the patient and two household members tested positive for adenovirus and rhinovirus.

**Laboratory results (38). **Thirty-five (95%; 37) patients received a positive conjunctival swab result, eight (28%; 29) patients received positive test results for combined nasal and oropharyngeal swabs, five (14%; 37) patients received positive nasopharyngeal swab test results, two (33%; 6) patients received positive nasal swab results, and one (25%; 4) patient received a positive oropharyngeal swab result (Table 2). The majority of patients had either a positive conjunctival or combined nasal/oropharyngeal swab (97%). One patient only received a positive nasal swab result with no other positive sites.

### Genetic Sequencing

Genetic sequencing of the viruses was performed from clinical specimens of 30 patients; all were identified as HPAI A(H5N1) clade 2.3.4.4b viruses. All eight gene segments of the viruses were recovered from 16 patients, and partial gene segments were recovered from the other 14. The viruses from the 16 patients with all gene segments sequenced (Figure) were identified as HPAI A(H5N1) clade 2.3.4.4b, genotype B3.13. The pediatric patient (A/California/192/2024) only had five of eight segments sequenced, which was insufficient to classify a specific genotype; however, the neuraminidase and nucleoprotein sequences shared close genetic identity to recent California HPAI A(H5N1) B3.13 genotype viruses from humans, dairy cattle, and poultry. One virus (A/California/150/2024) contained a nucleotide substitution within the polymerase acidic gene (I38M), which is associated with reduced susceptibility to the antiviral baloxavir marboxil.*** No substitutions associated with reduced oseltamivir susceptibility or adaptations for efficient human-to-human transmission were detected.

**FIGURE:**
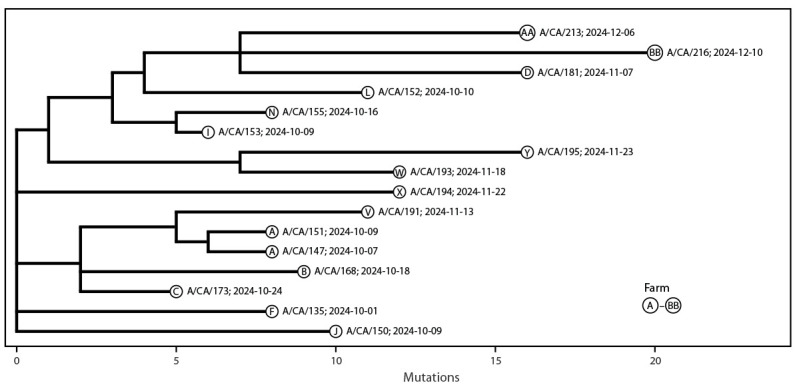
Phylogenetic tree* of 16 whole genome highly pathogenic avian influenza A(H5N1) viruses, by identification and collection date, from human cases — California, September–December 2024 * Tree was created with Ultrafast Sample placement on Existing tRee (UShER). https://genome.ucsc.edu/cgi-bin/hgPhyloPlace and Auspice https://auspice.us/

## Discussion

This report describes investigations that led to identification of 38 persons who received positive test results for HPAI A(H5N1) viruses in California; 37 were dairy farm workers with occupational exposure to sick cows, and one was a child aged <18 years with an undetermined exposure. Epidemiologic and clinical characteristics were similar to those in other U.S. human cases (*2*,*3*). In genetic sequencing of 30 of the 38 infected patients, all were identified as HPAI A(H5N1) clade 2.3.4.4b viruses. A substitution associated with reduced baloxavir susceptibility was identified in one virus sequenced from a human case in California. No additional concerning substitutions were identified.

The identification of 37 cases with occupational exposure across 29 dairy farms highlights the ongoing risk for cow-to-human transmission of HPAI A(H5N1) viruses among persons who have close contact with infected cows and their raw milk (*4*). The absence of cases among household contacts is consistent with the absence of viral genetic markers for efficient human-to-human transmission.

Although a majority of patients reported using PPE at work, use of recommended PPE (i.e., N95 respirators versus face mask) has been previously reported as being low among dairy farm workers with HPAI infection (*5*). Additional education and messaging about the risks of working with infected cows and ensuring worker access to PPE might increase PPE use, particularly if done in collaboration with farm worker organizations and producers.

This report describes the first detection of a pediatric case of influenza HPAI A(H5N1) in the United States. The source of this child’s infection remains undetermined. Unlike pediatric patients with HPAI A(H5N1) virus infections in other countries who had severe illness (*6*,*7*), this child had only mild respiratory symptoms and recovered quickly. Other sporadic cases of influenza HPAI A(H5N1) have occurred in persons with no known exposure to potentially infected animals (*8*). To date, human-to-human transmission of HPAI A(H5N1) viruses has not been identified in the United States.^†††^

### Limitations

The findings in this report are subject to at least three limitations. First, information about the type of and proportion of time that PPE was worn was unavailable for all patients. Second, access to PPE was not assessed. Finally, some symptomatic persons with exposure to sick animals might not have been reported, in which case some human HPAI A(H5N1) infections might have been missed.

### Implications for Public Health Practice

Public health agencies should work with dairy and poultry farms to reduce worker exposure to HPAI A(H5N1) viruses and detect and respond to human cases. Prevention, detection, and response strategies include PPE use guidance, training, and distribution; collaboration with farm managers on worker monitoring; working with LHDs to coordinate worker testing; specimen collection and laboratory testing to distinguish influenza A(H5) from seasonal influenza viruses; and distribution of oseltamivir treatment to HPAI A(H5N1) patients and oseltamivir prophylaxis to close contacts.^§§§^ Collaboration among public health, agriculture, animal health, occupational health, environmental health, health care providers, and other state and federal agencies is important for a coordinated One Health^¶¶¶^ response and to enable early detection of changes in influenza A(H5) viruses that could facilitate human-to-human transmission. Ongoing monitoring for genetic changes is necessary to assess the likelihood of antiviral resistance or human-to-human transmission of HPAI A(H5N1) viruses.

Expanded subtyping**** of influenza viruses might record additional cases of HPAI A(H5N1) virus infection with no known exposure (*8*). Health departments should evaluate potential exposures for all HPAI A(H5N1) cases to ascertain the possibility for human-to-human transmission. Surveillance for HPAI A(H5N1) viruses could include expanded subtyping for A(H5) testing in persons who meet epidemiologic and either clinical or public health criteria.

## Footnotes

*
https://ndc.services.cdc.gov/conditions/novel-influenza-a-virus-infections/

†
https://www.cdc.gov/bird-flu/signs-symptoms/index.html

§ CDC Human Influenza A/H5 Subtyping Kit (VER 4) Instructions for Use Package Insert. July 12, 2024.

¶
https://www.aphis.usda.gov/livestock-poultry-disease/avian/avian-influenza/hpai-detections/hpai-confirmed-cases-livestock

**
https://www.aphis.usda.gov/livestock-poultry-disease/avian/avian-influenza/hpai-detections/commercial-backyard-flocks

†† 45 C.F.R. part 46, 21 C.F.R. part 56; 42 U.S.C. Sect. 241(d); 5 U.S.C. Sect. 552a; 44 U.S.C. Sect. 3501 et seq.

§§
https://www.cdc.gov/bird-flu/prevention/worker-protection-ppe.html

¶¶
https://www.cdc.gov/bird-flu/spotlights/h5n1-response-12092024.html

***
https://www.cdc.gov/bird-flu/spotlights/h5n1-response-11152024.html

†††
https://www.cdc.gov/fluview/surveillance/2025-week-08.html

§§§
https://www.cdc.gov/bird-flu/prevention/hpai-interim-recommendations.html

¶¶¶
https://www.cdc.gov/one-health/php/about/index.html

****
https://www.cdc.gov/han/2025/han00520.html
